# Interstitial pneumonia with autoimmune features that met the proposed diagnostic criteria for IgG4‐related respiratory disease

**DOI:** 10.1002/rcr2.512

**Published:** 2019-12-17

**Authors:** Nobuhito Arakawa, Hideaki Yamasawa, Tamiko Takemura, Shinya Okada, Takafumi Taki, Shigemi Ishikawa

**Affiliations:** ^1^ Department of Pulmonary Medicine International University of Health and Welfare Hospital Nasushiobara Japan; ^2^ Department of Pathology Kanagawa Cardiovascular and Respiratory Center Yokohama Japan; ^3^ Department of Pathology International University of Health and Welfare Hospital Nasushiobara Japan; ^4^ Department of Chest Surgery International University of Health and Welfare Hospital Nasushiobara Japan; ^5^ Department of Thoracic Surgery Japan Community Health Care Organization Utsunomiya Hospital Utsunomiya Japan

**Keywords:** Hyperplasia of lymphoid follicles, IgG4‐related lung disease, interstitial pneumonia with autoimmune features, multidisciplinary discussion, pulmonary venous occlusion

## Abstract

We held a multidisciplinary discussion (MDD) about a 61‐year‐old woman who had an interstitial lung disease (ILD) without extrathoracic lesions that met the classification criteria for interstitial pneumonia with autoimmune features (IPAF) and the proposed diagnostic criteria for immunoglobulin G4 (IgG4)‐related respiratory disease (IgG4‐RRD). Clinically, the marked progression of lung‐limited diffuse lesions was consistent with IPAF. Serum IgG4 and rheumatoid factor levels simultaneously increased and did not contribute to a diagnosis. Pathologically, the significant hyperplasia of lymphoid follicles was consistent with rheumatoid arthritis (RA)‐associated ILD. Pulmonary venous occlusions by intimal fibrosis and intimal thickening were not important because these occlusions are found in IgG4‐related lung disease (IgG4‐RLD) and also in IPAF or ILDs related to connective tissue diseases (CTDs). Radiologically, fibrosing shadows that remained in the lung periphery after treatment were compatible with RA‐associated chronic ILD. We concluded that the present case was IPAF that met the proposed diagnostic criteria for IgG4‐RRD.

## Graphical abstract

We held a multidisciplinary discussion on a case of interstitial lung disease without extrathoracic lesions that met the classification criteria for interstitial pneumonia with autoimmune features (IPAF) and the proposed diagnostic criteria for immunoglobulin G4 (IgG4)‐related respiratory disease (IgG4‐RRD). We concluded that the present case was IPAF that met the proposed diagnostic criteria for IgG4‐RRD.

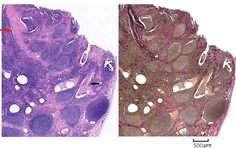

## Introduction

Interstitial pneumonia with autoimmune features (IPAF) is the term that was proposed in 2015 to describe idiopathic interstitial pneumonias (IIPs) with clinical features that imply some autoimmune background, but do not meet established criteria for connective tissue disease (CTD) 1.

Immunoglobulin G4‐related disease (IgG4‐RD) is a systemic fibroinflammatory disorder that is characterized by tumefactive lesions, the intensive infiltration of IgG4‐positive plasma cells, and storiform fibrosis and is generally accompanied by elevated serum IgG4 levels 2. The comprehensive diagnostic criteria for IgG4‐RD were established in 2011 3. As the development of lesions in each organ is not simultaneous, organ‐specific criteria were needed and accordingly established. Regarding respiratory organs, the “proposed diagnostic criteria for IgG4‐related respiratory disease” (hereinafter called “proposed IgG4‐RRD criteria”) were published in 2015 4.

We herein present a case of interstitial lung disease (ILD) diagnosed as IPAF that met the proposed IgG4‐RRD criteria after a detailed multidisciplinary discussion (MDD).

## Case Report

A 61‐year‐old woman developed a wet cough with dyspnoea in February 2018. She had a history of type 1 diabetes mellitus (DM) diagnosed in adolescence. She was not taking any medication other than insulin. She was a current smoker (20 cigarettes daily for 41 years). She had no history of alcohol consumption. She had worked as a cook in a restaurant with no history of dust inhalation. Her house was built 17 years earlier and received adequate sunshine. She had an ibuprofen allergy. Her brother had rheumatoid arthritis (RA) and her son had a history of neurosarcoidosis.

Although her wet cough attenuated over the course of one month, dyspnoea progressed. In April 2018, she visited a nearby hospital and an interstitial shadow was detected using chest computed tomography (CT). She was subsequently referred to our hospital.

**Figure 1:**
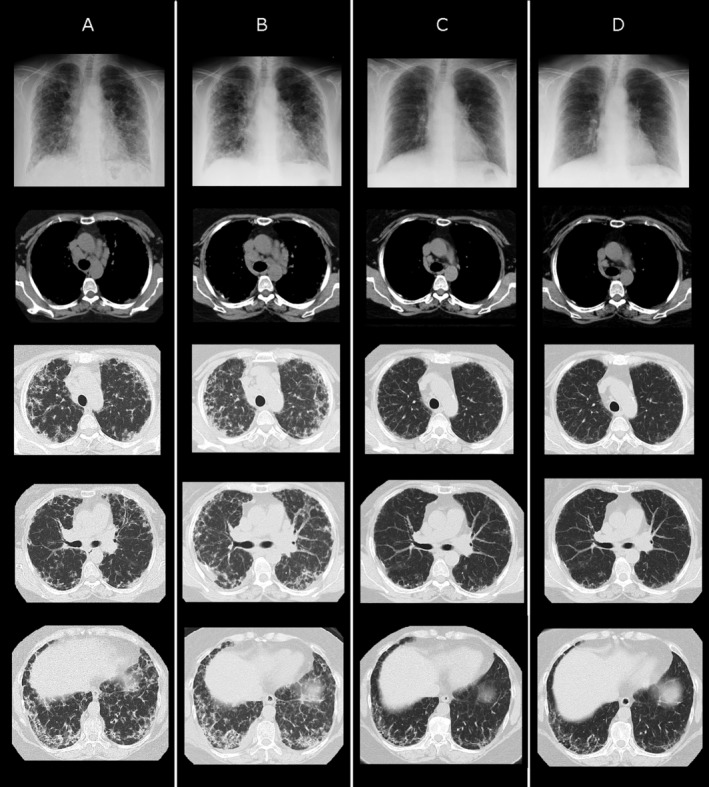
Time course of chest X‐ray and computed tomography. (A) On referral in April 2018. (B) At the initiation of treatment in the middle of August 2018. (C) Four months after the initiation of treatment (middle of December 2018). (D) One year after the initiation of treatment (August 2019).

Chest CT on referral (Fig. 1A) revealed diffuse bilateral interstitial shadows with bronchovascular bundle thickening, interlobular septal thickening, pleural thickening, and hilar and mediastinal lymphadenopathies. Our initial differential diagnoses were sarcoidosis, malignant lymphoma (ML), secondary ILD due to CTDs or drugs, and IgG4‐related lung disease (IgG4‐RLD). In a screening serological examination, serum IgG, IgG4, and rheumatoid factor (RF) levels were markedly elevated, and the Sjögren's syndrome type A antigen (SSA) antibody was positive (Table 1). ILD related to RA, Sjögren's syndrome, or IgG4‐RLD was suspected for differentiation. Whole‐body contrast CT and magnetic resonance imaging (MRI) of the head and neck were performed, and no affected lesions were observed, except for in the lung. She was admitted to our hospital for surgical lung biopsy (SLB) in July 2018.

**Table 1:** Laboratory findings on admission.

**Haematology**		γ‐Glutamyltransferase	22 U/L	Anti‐ARS antibody	Negative
White blood cell	10,920/μL	Amylase	129 U/L	MPO‐ANCA	Negative
Neutrophil	54.6%	C‐reactive protein	1.02 mg/dL	PR3‐ANCA	Negative
Lymphocyte	32.6%	KL‐6 (≤500 U/mL)	2900 U/mL		
Monocyte	5.5%	SP‐D (≤110 ng/mL)	314 ng/mL	**Arterial blood gas** (room air)	
Basophil	0.6%	sIL‐2R (122–496 U/mL)	1372 U/mL	pH	7.413
Eosinophil	6.7%	ACE (8.3–21.4 U/L)	19.9 U/L	PaCO_2_	36.7 Torr
Red blood cell	483×10^4^/μL	Interleukin‐6 (≤4 pg/mL)	6.9 pg/mL	PaO_2_	67.5 Torr
Haemoglobin	14.1 g/dL			HCO_3_^−^	23.0 mmol/L
Haematocrit	41.6%	**Immunology**			
Platelet	30.1×10^4^/μL	IgG	2436 mg/dL	**Pulmonary function** (% predicted)	
		IgG4	482 mg/dL	VC	1.93 L (81.4%)
		IgA	255 mg/dL	FVC	1.91 L (80.6%)
**Blood chemistry**		IgM	59 mg/dL	FEV_1_	1.67 L (89.8%)
Total protein	8.2 g/dL	Anti‐nuclear antibody	Negative	FEV_1_/FVC	87.4%
Albumin	3.5 g/dL	RF (≤15 IU/mL)	269 IU/mL	DLCO	8.87 mL/min/Torr (46.0%)
Blood urea nitrogen	20.6 mg/dL	C3	134 mg/dL	DLCO/VA	3.77 mL/min/Torr/L (80.7%)
Creatinine	0.88 mg/dL	C4	31 mg/dL		
AST	27 U/L	Anti‐CCP antibody	Negative	**6‐Min walking test** (room air)	
ALT	25 U/L	Anti‐SSA antibody	15.8 U/mL	SpO_2_ (0 → 6 min)	94% → 87%
LDH	271 U/L	Anti‐SSB antibody	Negative	Total distance	350 m
Total bilirubin	0.3 mg/dL	Anti‐RNP antibody	Negative		

ACE, angiotensin‐converting enzyme; ALT, alanine aminotransferase; ANCA, antineutrophil cytoplasmic antibodies; ARS, aminoacyl tRNA synthetase; AST, aspartate aminotransferase; CCP, cyclic citrullinated peptide; DLCO, diffusing capacity of the lungs for carbon monoxide; DLCO/VA, DLCO divided by the alveolar volume; FEV_1_, forced expiratory volume in 1 sec; FVC, forced vital capacity; Ig, immunoglobulin; KL‐6, Krebs von den Lungen‐6; LDH, lactate dehydrogenase; MPO, myeloperoxidase; PaCO_2_, partial pressure of arterial carbon dioxide; PaO_2_, partial pressure of arterial oxygen; PR3, proteinase 3; RF, rheumatoid factor; RNP, ribonucleoprotein; sIL‐2R, soluble interleukin‐2 receptor; SP‐D, surfactant protein‐D; SpO_2_, arterial oxygen saturation of pulse oxymetry; SSA, Sjögren's syndrome type A antigen; SSB, Sjögren's syndrome type B antigen; VC, vital capacity.

On admission, her height was 151 cm and weight was 70 kg. She had no fever and her resting respiratory rate was 16/min; however, she had distinct dyspnoea on effort. Her fingers were clubbed, and fine crackles were audible in the lower chest. There were no palpable surface lymph nodes. She had neither arthralgia nor skin lesions that suggested CTDs.

**Figure 2:**
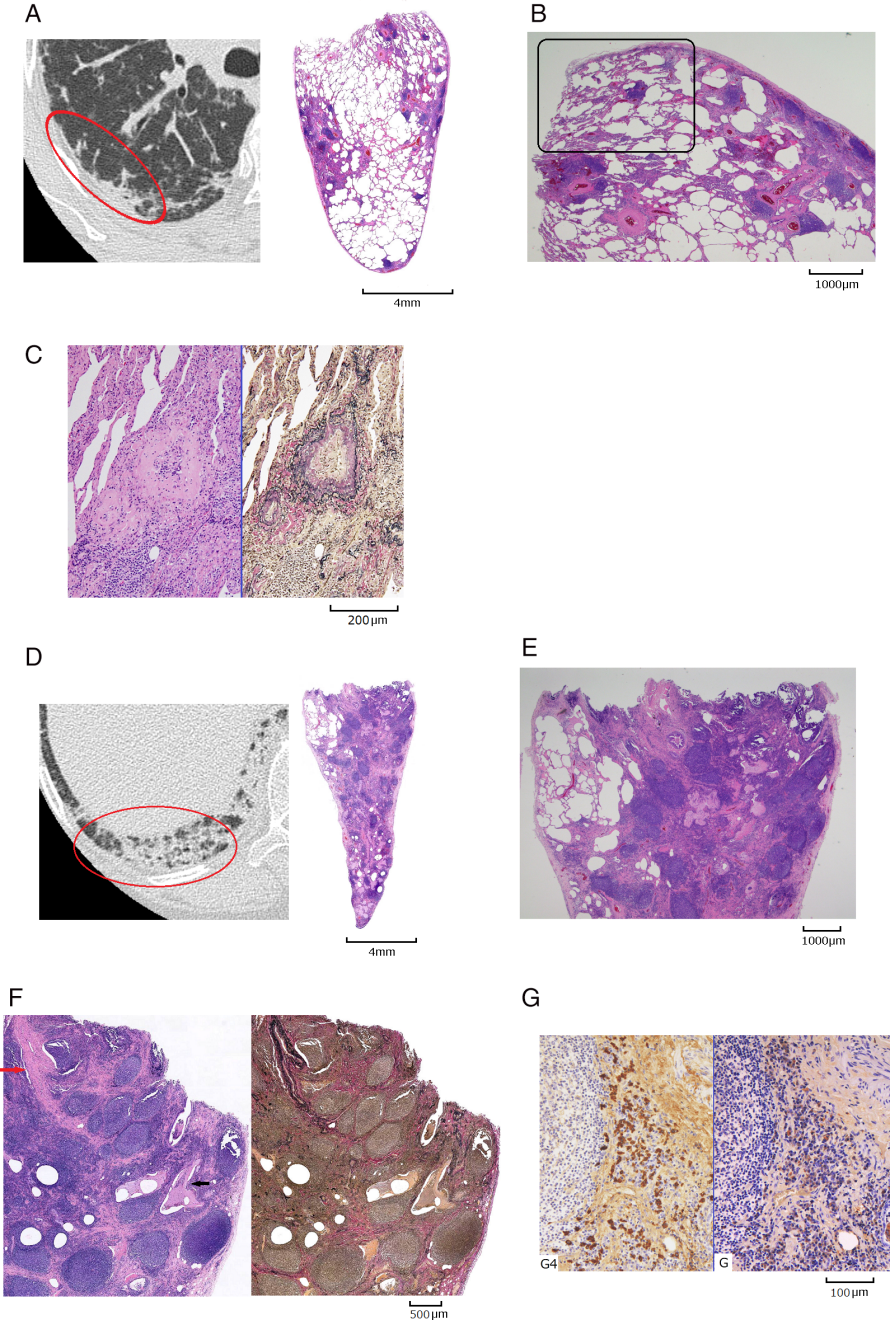
HRCT of surgically resected portions taken one week prior to biopsy (red circles) and histological findings (right upper lobe S2 (A–C)and right lower lobe S10 (D–G)). (A) The hyperplasia of lymphoid follicles and infiltration of lymphoplasmacytes were observed in peribronchovascular bundles, the interlobular septum, and subpleural areas (left, HRCT; right, HE; loupe). (B) Destruction of the alveolar structure was limited and the distribution of fibrosis was uneven. The NSIP pattern was observed in some portions (black frame) (HE, original magnification 1.25×). (C) Venous occlusion by intimal fibrosis and intimal thickening with the infiltration of lymphocytes and plasma cells (left, HE; right, EVG; original magnification 10×). (D) The hyperplasia of lymphoid follicles and infiltration of lymphoplasmacytes were observed in peribronchovascular bundles, the interlobular septum, and subpleural areas (left, HRCT; right, HE; loupe). (E) Destruction of the alveolar structure was more intense in the right lower lobe specimen than in the right upper lobe specimen (HE, original magnification 1.25×). (F) Fibrotic changes and the hyperplasia of lymphoid follicles with an enlarged germinal centre were extensive. The peribronchiolar infiltration of lymphocytes and plasma cells resulted in the loss of the bronchiolar elastic lamina and destruction of the bronchiolar wall. The lumen of a bronchiole was enlarged to show a cystic appearance (black arrow). Septal veins were partially occluded by intimal fibrosis and intimal proliferation (red arrow). (left, HE; right, EVG; original magnification 4×) (G) The IgG4 stain (left) and IgG stain (right) show an IgG4/IgG cell ratio of 56%, and the IgG4‐positive cell count was 159 cells in an HPF (original magnification 10×). HRCT, high‐resolution computed tomography; EVG, elastica van Gieson; HE, haematoxylin–eosin; HPF, high‐power field; Ig, immunoglobulin; NSIP, non‐specific interstitial pneumonia.

SLB was performed by video‐assisted thoracoscopic surgery (VATS). Specimens were taken from a radiologically less affected part and an intensively affected part; the right upper lung (S2) and the right lower lung (S10), respectively (Fig. 2A, D). In the S2 and S10 specimens, the hyperplasia of lymphoid follicles with a germinal centre and the infiltration of lymphoplasmacytic cells were observed in bronchovascular bundles, the interlobular septum, and subpleural areas (Fig. 2B, E). The hyperplasia of lymphoid follicles, the infiltration of lymphoplasmacytic cells, and destruction of the alveolar structure were more intense in the S10 specimen. In some areas, marked bronchiolitis with destruction of its walls, resulting in cystic changes, was observed (Fig. 2F). No obvious lymphoepithelial lesions were found from the bronchioles to alveoli, and an intact follicular structure was confirmed by immunostaining to exclude malignancy. In the S2 specimen, the non‐specific interstitial pneumonia (NSIP) pattern was observed in some portions (Fig. 2B). These results were pathologically consistent with RA‐related ILD 5, but clinically did not meet the RA classification criteria 6. Furthermore, as the SSA antibody was positive, lip biopsy was performed, and Sjögren's syndrome was ruled out. Based on the results obtained, the present case serologically and morphologically met the classification criteria for IPAF 1 (hereinafter called “IPAF criteria”).

Regarding the possibility of IgG4‐RLD, in the S10 specimen, the IgG4/IgG cell ratio was 56%, and the IgG4‐positive cell count was 159 cells in a high‐power field (HPF) (Fig. 2G). Some pulmonary veins were occluded by intimal fibrosis and intimal thickening with the infiltration of lymphocytes and plasma cells in both the S2 and S10 specimens, which was consistent with obliterative phlebitis (Fig. 2C, F). These histological findings, CT findings, and serology met definite IgG4‐RLD under the proposed IgG4‐RRD criteria, although extrathoracic lesions were absent.

Since it took four months from her referral to the initiation of treatment, her interstitial shadows and dyspnoea had progressed (Fig. 1B). However, oral prednisolone (PSL) at 30 mg/day started in the middle of August 2018 was very effective, and was successfully tapered to 10 mg/day in November 2018. Her dyspnoea and interstitial shadows markedly improved, although fibrosing shadows remained in the lung periphery (Fig. 1C). In May 2019, PSL was reduced to 7.5 mg/day and no recurrence was observed in August 2019 (Fig. 1D).

### Discussion

We herein report a case of ILD without extrathoracic lesions that met both the IPAF criteria and the proposed IgG4‐RRD criteria. As difficulties were associated with differentiating between these two conditions, we held an MDD.

Clinically, IPAF was more likely than IgG4‐RLD without extrathoracic lesions because it was unusual to detect no extrathoracic IgG4‐related lesions despite the marked progression of extensively diffuse pulmonary lesions. This progression of lung‐limited diffuse lesions was acceptable for IPAF. IgG4‐RLD without extrathoracic lesions is very rare 7.

Serologically, difficulties were associated with distinguishing IPAF from IgG4‐RLD without extrathoracic lesions because elevated serum IgG4 levels are not specific to IgG4‐RD; they are also often elevated in CTDs, particularly in RA and Churg–Strauss syndrome (CSS) 8. Alternatively, RF levels are frequently elevated in IgG4‐RD 9. It is important to note that serum IgG4 and RF levels may simultaneously become elevated, and, thus, may not contribute to a diagnosis in cases such as that described herein.

Pathologically, IPAF was more likely than IgG4‐RLD without extrathoracic lesions because the significant hyperplasia of lymphoid follicles in the present case is sometimes detected in RA‐related ILD 5, but is unusual for IgG4‐RLD 10. It is important to note that pulmonary venous occlusions by intimal fibrosis and intimal thickening did not contribute to reaching a diagnosis in the present case; this type of venous occlusion can be found in CTDs with pulmonary arterial hypertension (PAH) as well as in IgG4‐RD. Paradoxically, in CTDs with PAH, obstructive pulmonary vascular lesions are predominantly observed in the veins and venules, the lumens of which are occluded by loose intimal fibrosis with inflammatory cells 11. Even with a latent degree of PAH due to CTDs, this pulmonary venous alteration may be expected. On the other hand, venous occlusion or obliterative phlebitis, which has diagnostic value for IgG4‐RD, is occlusion by lymphoplasmacytic infiltration and fibrosis against a loose textured background with diameter of more than 150 μm 12. As these pulmonary venous occlusions may have a similar appearance, they are not useful for distinguishing IgG4‐RLD from IPAF or CTD‐related ILDs.

Radiologically, it was more reasonable to diagnose the case as IPAF than IgG4‐RLD without extrathoracic lesions. Chest CT before steroid therapy (Fig. 1A, B) revealed diffuse interstitial shadows with bronchovascular bundle thickening, interlobular septal thickening, pleural thickening, and hilar and mediastinal lymphadenopathies. These radiological findings may be found in both IgG4‐RLD 4 and RA‐associated ILD 13. The point is the fibrosing shadows that remained in the lung periphery after the glucocorticoid treatment (Fig. 1C, D). These steroid‐resistant fibrosing shadows are compatible with RA‐associated chronic ILD and incompatible with IgG4‐RLD.

We concluded that the present case was IPAF that met the proposed IgG4‐RRD criteria. The present case suggests that the concept of IgG4‐RRD needs more refinements. We should proceed with investigation into pathogenesis of the disease. The present case also reveals that we need further study to clarify the relationship between IPAF and IgG4.

### Disclosure Statement

Appropriate written informed consent was obtained for publication of this case report and accompanying images.

This case was presented and discussed at the 20th Tokyo Diffuse Lung Diseases Study Conference held in Tokyo on 26 October 2019 under the joint sponsorship of the Tokyo Diffuse Lung Diseases Study Group and Shionogi Pharmaceutical Co., Ltd. I, Nobuhito Arakawa, declare on behalf of my co‐authors and myself that we have no financial relationship with any commercial entity having an interest in the subject of this manuscript.
